# Association of a Multisite Interprofessional Education Initiative With Quality of Primary Care

**DOI:** 10.1001/jamanetworkopen.2019.15943

**Published:** 2019-11-20

**Authors:** Samuel T. Edwards, Elizabeth R. Hooker, Rebecca Brienza, Bridget O’Brien, Hyunjee Kim, Stuart Gilman, Nancy Harada, Lillian Gelberg, Sarah Shull, Meike Niederhausen, Samuel King, Elizabeth Hulen, Mamta K. Singh, Anaïs Tuepker

**Affiliations:** 1 Section of General Internal Medicine, Veterans Affairs Portland Health Care System, Portland, Oregon; 2 Department of Family Medicine, Oregon Health and Science University, Portland; 3 Center to Improve Veteran Involvement in Care, Veterans Affairs Portland Health Care System, Portland, Oregon; 4 Division of General Internal Medicine and Geriatrics, Department of Medicine, Oregon Health and Science University, Portland; 5 Center of Excellence in Primary Care Education, Veterans Affairs Connecticut Health Care System, West Haven; 6 Department of Internal Medicine, Yale University School of Medicine, New Haven, Connecticut; 7 Center of Excellence in Primary Care Education, Veterans Affairs San Francisco Health Care System, San Francisco, California; 8 Department of Medicine and Office of Medical Education, University of California, San Francisco; 9 Center for Health Systems Effectiveness, Oregon Health and Science University, Portland; 10 Office of Academic Affiliations, Veterans Health Administration, Washington, DC; 11 David Geffen School of Medicine, University of California, Los Angeles; 12 Center of Excellence in Primary Care Education, Veterans Affairs Greater Los Angeles Health Care System, Los Angeles, California; 13 Oregon Health and Science University–Portland State University School of Public Health, Oregon Health and Science University, Portland; 14 Center of Excellence in Primary Care Education, Louis Stokes Cleveland Veterans Affairs Medical Center, Cleveland, Ohio; 15 Case Western University School of Medicine, Cleveland, Ohio

## Abstract

**Importance:**

Studies have shown that interprofessional education (IPE) improves learner proficiencies, but few have measured the association of IPE with patient outcomes, such as clinical quality.

**Objective:**

To estimate the association of a multisite IPE initiative with quality of care.

**Design, Setting, and Participants:**

This study used difference-in-differences analysis of US Department of Veterans Affairs (VA) electronic health record data from July 1, 2008, to June 30, 2015. Patients cared for by resident clinicians in 5 VA academic primary care clinics that participated in the Centers of Excellence in Primary Care Education (CoEPCE), an initiative designed to promote IPE among physician, nurse practitioner, pharmacist, and psychologist trainees, were compared with patients cared for by resident clinicians in 5 regionally matched non-CoEPCE clinics using data for the 3 academic years (ie, July 1 to June 30) before and 4 academic years after the CoEPCE launch. Analysis was conducted from January 18, 2018, to January 17, 2019.

**Main Outcomes and Measures:**

Among patients with diabetes, outcomes included annual hemoglobin A_1c_, poor hemoglobin A_1c_ control (ie, <9% or unmeasured), and annual renal test; among patients 65 years and older, outcomes included prescription of high-risk medications; among patients with hypertension, outcomes included hypertension control (ie, blood pressure, <140/90 mm Hg); and among all patients, outcomes included timely mental health referrals, primary care mental health integrated visits, and hospitalizations for ambulatory care–sensitive conditions.

**Results:**

A total of 44 527 patients contributed 107 686 patient-years; 49 279 (45.8%) were CoEPCE resident patient-years (mean [SD] patient age, 59.3 [15.2] years; 26 206 [53.2%] white; 8073 [16.4%] women; mean [SD] patient Elixhauser comorbidity score, 12.9 [15.1]), and 58 407 (54.2%) were non-CoEPCE resident patient-years (mean [SD] patient age, 61.8 [15.3] years; 43 912 [75.2%] white; 4915 [8.4%] women; mean [SD] patient Elixhauser comorbidity score, 13.8 [15.7]). Compared with resident clinicians who did not participate in the CoEPCE initiative, CoEPCE training was associated with improvements in the proportion of patients with diabetes with poor hemoglobin A_1c_ control (−4.6 percentage points; 95% CI, −7.5 to −1.8 percentage points; *P* < .001), annual renal testing among patients with diabetes (3.2 percentage points; 95% CI, 0.6 to 5.7 percentage points; *P* = .02), prescription of high-risk medications among patients 65 years and older (−2.3 percentage points; 95% CI, −4.0 to −0.6 percentage points; *P* = .01), and timely mental health referrals (1.6 percentage points; 95% CI, 0.6 to 2.6 percentage points; *P* = .002). Fewer patients cared for by CoEPCE resident clinicians had a hospitalization for an ambulatory care–sensitive condition compared with patients cared for by non-CoEPCE resident clinicians in non-CoEPCE clinics (−0.4 percentage points; 95% CI, −0.9 to 0.0 percentage points; *P* = .01). Sensitivity analyses with alternative comparison groups yielded similar results.

**Conclusions and Relevance:**

In this study, the CoEPCE initiative was associated with modest improvements in quality of care. Implementation of IPE was associated with improvements in patient outcomes and may potentiate delivery system reform efforts.

## Key Points

**Question:**

Is the implementation of an interprofessional education initiative in US Department of Veterans Affairs primary care clinics associated with changes in quality of care?

**Findings:**

In this study using difference-in-differences analysis of Department of Veterans Affairs electronic health record data, patients cared for by resident clinicians who participated in a large, multisite, interprofessional education quality improvement initiative had modestly improved quality of care compared with patients cared for by resident clinicians at similar, nonparticipating Department of Veterans Affairs teaching clinics.

**Meaning:**

In this study, interprofessional education in primary care was associated with improvements in quality of care.

## Teaser

This study estimates the association of a multisite interprofessional education initiative in the US Department of Veterans Affairs primary care clinics with quality of care.

## Introduction

With the increasing complexity of patients and the health care system that serves them, interdisciplinary, team-based approaches are needed for effective care.^1^ Patients often require the expertise of different professional disciplines to be integrated and coordinated by high-functioning, collaborative teams. However, health professionals train in silos with distinct professional cultures, perpetuating hierarchical structures and limiting development of skills in collaborative practice. Thus, it has been argued that efforts to implement team-based care models will have limited success without coincident educational reforms that create clinical learning environments to teach collaborative skills.^2,3^

Interprofessional education (IPE), or the activity of 2 or more professions learning about, from, and with each other, is an approach put forth by policy makers to support the development of a workforce competent in team-based care.^4,5^ Research shows that IPE can change learners’ attitudes toward interprofessional care and enhance collaborative knowledge and skills.^6,7^ Additionally, randomized clinical trials of IPE interventions have demonstrated improvements in team behaviors,^8,9,10^ patient-centered communication,^11^ patient satisfaction,^12^ and clinical work processes.^12,13,14^ The inclusion of multiple professions in the management of chronic disease has also been associated with improved outcomes.^15,16,17,18^ However, few studies have evaluated the effect of IPE on clinical outcomes, and those published have small sample sizes, short time frames, and mixed results.^8,19,20,21,22^

In 2011, the US Department of Veterans Affairs (VA) Office of Academic Affiliations (OAA) launched the Centers of Excellence in Primary Care Education Initiative (CoEPCE) to promote the IPE of physicians, nurse practitioners (NPs), psychologists, and pharmacists in 5 primary care teaching sites. The CoEPCE initiative coincided with the VA’s national implementation of Patient Aligned Care Teams (PACT), a patient-centered, medical home primary care model that included the establishment of interprofessional teams.^23^ Hence, the CoEPCE initiative was conceived as a potentially necessary educational reform for the long-term success of the PACT model of care. The CoEPCE initiative included alignment of trainee schedules, colocated didactics and clinical experiences, collaborative quality improvement projects, shared responsibility for clinical care among trainees from multiple professions, and a shift from didactic instruction to supervised clinical experiences.^24^ This study aimed to estimate the association of the CoEPCE initiative with quality of care among patients cared for by interprofessional trainees in the context of the implementation of PACT.

## Methods

### Overview

This study is part of the Interprofessional Learning and Practice Partnered Evaluation Center, funded by the VA Quality Enhancement Research Initiative and OAA to perform a longitudinal, mixed-methods evaluation of the CoEPCE initiative. The Veterans Health Administration determined this work to be a quality improvement activity, with a waiver of informed consent. This report follows Standards for Quality Improvement Reporting Excellence (SQUIRE) reporting guideline.

### Intervention

The CoEPCE initiative was a coordinated initiative within the VA designed to develop and test innovative approaches for curricula for health profession trainees related to core competencies of patient-centered care and to study the effect of new educational approaches and models on health profession education to include collaboration, cultural shifts in educational priorities, and educational, clinical, and workforce outcomes within and beyond VA. In 2010, OAA announced a request for proposals for VA facilities to seek funding to develop and implement interprofessional team-based curricula to achieve clinical practice and education integration. Requirements included partnerships with academic affiliates, inclusion of physician residents and NP students; plans to incorporate other professions when resources and expertise became available; curriculum development focused on 4 core educational domains (ie, shared decision-making, interprofessional collaboration, sustained relationships, and performance improvement); and the use of workplace learning as an instructional strategy. Site staffing requirements included leadership teams consisting of a physician and NP codirector and faculty including at least 4 clinician educators with protected time to fulfill curriculum development, teaching, and mentoring responsibilities.^24^

In 2011, 5 VA facilities were selected to participate, as follows: Boise, Idaho; Cleveland, Ohio; San Francisco, California; Seattle, Washington; and West Haven, Connecticut. Training activities began in July 2011. Initially, the CoEPCE sites included primary care NP students and/or residents and internal medicine physician residents, but the program was later expanded to include health psychology, pharmacy, social work, and physician assistant trainees. Given contextual differences among sites (eg, number of trainees, faculty expertise, space, access to supplemental resources), sites designed and implemented curricula in different ways, although each addressed the same 4 domains. Components included aligning trainee schedules, designing joint didactics, and running interprofessional patient care and quality improvement activities. Over time, sites learned from their own and other sites’ experiences, which led to some convergence of intervention approaches. Components of the CoEPCE initiative are described in detail elsewhere.^24^

### Study Design

We performed a quality improvement study comparing clinical outcomes among primary care patients cared for by CoEPCE resident clinicians with outcomes among patients cared for by resident clinicians in regionally matched, non-CoEPCE academic primary care PACT clinics. We used a difference-in-differences approach to compare changes in outcomes 3 years before the CoEPCE initiative launch (ie, July 1, 2008, to June 30, 2011) to 4 years after CoEPCE launch (July 1, 2011, to June 30, 2015) between CoEPCE and comparison groups.

### Population

We defined patient-year cohorts by academic year from 2008 (ie, July 1, 2008, to June 30, 2009) through 2014 (ie, July 1, 2014, to June 30, 2015).^25^ While the CoEPCE initiative included multiple professions, interprofessional CoEPCE trainees typically worked with patients assigned to CoEPCE resident clinicians (ie, internal medicine or NP residents). Hence, patients were included in the CoEPCE group if they were assigned to a CoEPCE primary care team at a CoEPCE site and assigned only to an internal medicine or NP resident as their primary care practitioner during the measurement year. Team assignments had to be present for more than 60% of assigned time in the measurement year, and patients had to have had at least 1 primary care visit in that year. Patients were included in the comparison group if they were assigned to an academic PACT team at a non-CoEPCE site and only to an internal medicine or NP resident for more than 60% of the assigned time in the measurement year with at least 1 primary care visit in that year.

We selected 5 VA sites as comparison sites. Comparison sites were matched by region, had similar facility complexity, and had the same health profession training programs present as CoEPCE sites (ie, internal medicine residents, NP trainees, psychology trainees, and pharmacy students). Facility complexity was assessed in fiscal year 2011 (ie, October 1, 2010, to September 30, 2011) using the Veterans Health Administration Facility Complexity Model, which is based on patient population, clinical services complexity, and facility participation in education and research.^26^ We contacted VA personnel at comparison sites to ensure the presence of appropriate professional training programs. The sites chosen to serve as our comparison group were Palo Alto, California; Pittsburgh, Pennsylvania; Portland, Oregon; Providence, Rhode Island; and Salt Lake City, Utah.

### Study Variables

On study initiation, the CoEPCE sites, OAA, and the Interprofessional Learning and Practice Partnered Evaluation Center created a working group that developed a core set of outcome measures that curricula and educational activities at each CoEPCE site would address (eg, through quality improvement projects, collaborative case conferences, or panel management activities) and thus could, in theory, be affected by the CoEPCE initiative. Outcomes included measures that could be affected by improved interprofessional teamwork, such as team-based panel management, and those that would benefit from expertise from specific professions. Outcomes included 3 measures of diabetes care quality, as follows: having an annual hemoglobin A_1c_ (HbA_1c_) test, having poor HbA_1c_ control (ie, >9% of total hemoglobin or unmeasured), and annual renal testing (ie, urinary microalbumin, prescription of an angiotensin-converting enzyme inhibitor, or prescription of an angiotensin receptor blocker), and 1 measure of hypertension control (blood pressure, <140/90 mm Hg). We included a measure of high-risk medication use in older patients (ie, ≥65 years) using the 2015 Beers criteria to capture pharmacists’ potential contributions to the initiative.^27^ To reflect the initiative’s inclusion of psychologists on interprofessional teams and the clinical focus on primary care mental health integration, we developed a measure of timely mental health visits (ie, mental health visit within 24 hours of a primary care visit). We also measured use of integrated primary care mental health visits, a specific visit type in which mental health clinicians see patients in primary care clinics. As we were interested in the possible substitution of primary care use for hospital use, we included hospitalizations for ambulatory care–sensitive conditions (ACSCs).^28^ We also extracted data from the VA’s electronic health record on patient age, sex, race/ethnicity, and comorbidities for use as covariates. All data were extracted for each measurement year from the VA Corporate Data Warehouse. Elixhauser comorbidities were calculated for each measurement year with a 2-year look-back period.

### Statistical Analysis

We compared patient-year characteristics between CoEPCE and comparison groups using descriptive statistics. To estimate changes in quality of care associated with the CoEPCE initiative, we estimated a difference-in-differences patient-year level model. The design controlled for differences between CoEPCE and non-CoEPCE sites that existed before the implementation of the CoEPCE initiative as well as time trends that reflected broader health care changes among the patient population. All models were adjusted for age, sex, race/ethnicity, Elixhauser comorbidity^29^ score, and years in VA care. We included site as a random effect, using random intercepts whose error was modeled using a normal distribution with an identity covariance matrix structure to account for the correlation among patients within site. All covariates were calculated for each measurement year. We included indicators for CoEPCE group, the postintervention period, and the interaction between the CoEPCE group and postintervention period, which provided estimates of CoEPCE effects. Our outputs were the estimated probabilities and counts for each group in the preintervention and postintervention periods, the change between preintervention and postintervention periods for each group, and the differences between the 2 groups’ change (ie, the difference-in-differences). A total of 5 models had analytic samples that were restricted to patients who were eligible for that outcome (ie, diagnosis of diabetes: annual HbA_lc_, poor HbA_1c_ control, and annual renal test; diagnosis of hypertension: hypertension control; and patients aged ≥65 years: high-risk medication use). All other models included the full sample.

We used logistic mixed-effects models and estimated average marginal effects for straightforward interpretation of the association of the CoEPCE initiative with outcomes (eMethods in the Supplement). We also examined the parallel trend assumption by modeling and examining line plots of the 2 groups in the preintervention period for all included outcomes.

**Figure 1.**
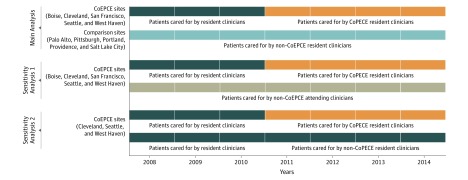
Cohort Construction for Main Analysis and Sensitivity Analyses The main analysis compared patients cared for by Centers of Excellence of Primary Care Education (CoEPCE) resident clinicians at CoEPCE sites with patients cared for by resident clinicians at non-CoEPCE sites. In sensitivity analysis 1, patients cared for by CoEPCE resident clinicians were compared with patients cared for by attending clinicians from the same CoEPCE sites. Sensitivity analysis 2 was conducted at 3 sites that divided their resident clinicians into 2 groups, some who participated in the CoEPCE initiative and some who did not. Patients cared for by CoEPCE resident clinicians were compared with patients cared for by non-CoEPCE resident clinicians from within these 3 sites.

A threat to the validity of our analysis was that other differences between the CoEPCE sites and non-CoEPCE comparison sites may have influenced quality of care over time, affecting our estimates of the association of the CoEPCE initiative with outcomes. To account for this, we constructed 2 alternative comparison groups of patients drawn from CoEPCE clinics (Figure 1). We then constructed analogous models comparing outcomes among CoEPCE patients to outcomes among these groups.

The first alternative comparison group included patients cared for by attending clinicians at CoEPCE sites. In all 5 CoEPCE sites, attending clinicians (who supervised clinician trainees) maintained separate patient panels within the same clinics, and these attending clinicians and their patients were not part of the CoEPCE initiative. We constructed a cohort of patients cared for by these attending clinicians.

The second alternative comparison group included patients cared for by resident clinicians who did not participate in the CoEPCE initiative but did train at CoEPCE sites. In 3 of 5 CoEPCE sites (ie, Cleveland, Seattle, and West Haven), resident clinicians were divided into 2 groups: approximately half participated in the CoEPCE initiative, and approximately half did not. At these 3 sites we identified patients cared for by CoEPCE participant resident clinicians and patients cared for by nonparticipating resident clinicians and compared outcomes between these groups.

We used a 2-sided *P* < .05 as a significance threshold. Analyses were performed in Stata version 15 (StataCorp). Analyses were conducted from January 18, 2018, to January 17, 2019.

## Results

We identified a total of 44 527 patients who contributed 107 686 patient-years. We compared outcomes of patients cared for by resident clinicians at CoEPCE sites (49 279 [45.8%] patient-years; 24 218 [22.5%] before the intervention and 25 061 [23.3%] after the intervention) to outcomes of patients cared for by resident clinicians at non-CoEPCE sites (58 407 patient-years; 23 281 [21.6%] before the intervention and 35 126 [32.6%] after the intervention). Patient-year characteristics are presented in Table 1. Patient-years at CoEPCE sites corresponded to 26 206 (53.2%) white individuals and 8073 (16.4%) women, with a mean (SD) age of 59.3 (15.2) years and a mean (SD) Elixhauser comorbidity score of 12.9 (15.1). Non-CoEPCE patient-years were similar in mean age and comorbidity score (ie, mean [SD] age, 61.8 [15.3] years; mean [SD] Elixhauser comorbidity score, 13.8 [15.7]) but had a lower proportion of women (4915 [8.4%]) and a higher proportion of white patients (43 912 [75.2%]).

**Table 1.** Patient-Year Characteristics of Patients Cared for by Resident Clinicians at 5 CoEPCE Clinics vs Patients Cared for by Resident Clinicians at 5 Non-CoEPCE Clinics

Characteristic	No. (%)		*P* Value
	Patients of Non-CoEPCE Resident Clinicians (n = 58 407)	Patients of CoEPCE Resident Clinicians (n = 49 279)	
Age, mean (SD), y	61.8 (15.3)	59.3 (15.2)	<.001
Women	4915 (8.4)	8073 (16.4)	<.001
Race/ethnicity			
White	43 912 (75.2)	26 206 (53.2)	<.001
Black	6522 (11.2)	13 257 (26.9)	
Hispanic	2224 (3.8)	1637 (3.3)	
Other or unknown	5749 (9.8)	8179 (16.6)	
Medically complex^a^	7648 (13.1)	5675 (11.5)	<.001
VA care, y			
Mean (SD)	7.7 (4.5)	7.4 (4.6)	<.001
<5	19 371 (33.2)	17 416 (35.3)	<.001
5-10	17 855 (30.6)	15 416 (31.3)	
>10	21 181 (36.3)	16 447 (33.4)	
Elixhauser comorbidity score			
Mean (SD)	13.8 (15.7)	12.9 (15.1)	<.001
Median (IQR) [range]	9 (0 to 21) [–4 to 148]	9 (0 to 20) [–4 to 117]	
Selected Elixhauser comorbidities			
Congestive heart failure	4254 (7.3)	2748 (5.6)	<.001
Hypertension	34 293 (58.7)	27 851 (56.5)	<.001
Chronic pulmonary disease	8864 (15.2)	6894 (14.0)	<.001
Diabetes without complications	10 335 (17.7)	8252 (16.7)	<.001
Diabetes with chronic complications	5230 (9.0)	3017 (6.1)	<.001
Hypothyroidism	4101 (7.0)	2651 (5.4)	<.001
Renal failure	5250 (9.0)	3294 (6.7)	<.001
Liver disease	3209 (5.5)	3056 (6.2)	<.001
Metastatic cancer	541 (0.9)	444 (0.9)	.66
Obesity	9920 (17.0)	7897 (16.0)	<.001
Alcohol use disorder	6737 (11.5)	6681 (13.6)	<.001
Drug use disorder	4507 (7.7)	5126 (10.4)	<.001
Psychoses	9361 (16.0)	8105 (16.4)	.06
Depression	12 879 (22.1)	9381 (19.0)	<.001

Abbreviations: CoEPCE, Centers of Excellence in Primary Care Education; IQR, interquartile range; VA, Veterans Affairs.

^a^ Includes patients whose Elixhauser Comorbidity scores were in at least the 90th percentile.

Results from difference-in-differences analyses are presented in Table 2. For 5 of 8 measures, we found the CoEPCE initiative associated with improvements among patients cared for by CoEPCE resident clinician vs patients cared for by non-CoEPCE resident clinicians in non-CoEPCE clinics, before and after the CoEPCE initiative launch. Patients who were cared for by CoEPCE resident clinicians were associated with improvements in HbA_1c_ control (patients with poor HbA_1c_ control, −4.6 percentage points; 95% CI, −7.5 to −1.8 percentage points; *P* = .001), proportion of patients with diabetes with annual renal testing (3.2 percentage points; 95% CI, 0.6 to 5.7 percentage points; *P* = .02), proportion of patients 65 years or older receiving a high-risk medication (−2.3 percentage points; 95% CI, −4.0 to −0.6 percentage points; *P* = .01), and proportion of patients who had a timely mental health referral (1.6 percentage points; 95% CI, 0.6 to 2.6 percentage points; *P* = .002). Fewer patients cared for by CoEPCE resident clinicians had hospitalizations for an ACSC (−0.4 percentage points; 95% CI, −0.9 to 0.0 percentage points; *P* = .01). For 3 of 8 measures, there were no significant difference-in-differences (annual A_1c_ testing: 0.7 percentage points; 95% CI, −0.7 to 2.1; *P* = .37; hypertension control: −0.5 percentage points; 95% CI, −3.7 to 2.7; *P* = .77; primary care mental health integrated visits: −0.1; 95% CI, −0.9 to 0.8; *P* = .045). Our models met the difference-in-difference assumption for parallel trend, with *P* > .05 in preintervention models (eTable 1 in the Supplement). Models that did not meet the assumption for parallel trend are presented in eTable 2 in the Supplement.

**Table 2.** Changes in Quality of Care Measures and Health Care Utilization Among Patients of CoEPCE Resident Clinicians and Patients of Non-CoEPCE Resident Clinicians Before and After Initiative Launch

Outcome	Estimated Probability (95% CI)^a^								Difference in Differences	
	Patients of Non-CoEPCE Resident Clinicians				Patients of CoEPCE Resident Clinicians					
	2008-2010	2011-2014	Difference	*P* Value	2008-2010	2011-2014	Difference	*P* Value	Difference	*P* Value
Annual HbA_1c _test	0.960 (0.951 to 0.968)	0.962 (0.955 to 0.969)	0.002 (–0.007 to 0.012)	.61	0.952 (0.942 to 0.961)	0.961 (0.952 to 0.969)	0.009 (–0.002 to 0.020)	.10	0.007 (–0.007 to 0.021)	.37
Poor HbA_1c_ control	0.194 (0.177 to 0.212)	0.233 (0.216 to 0.250)	0.039 (0.020 to 0.058)	<.001	0.234 (0.214 to 0.253)	0.226 (0.207 to 0.245)	–0.007 (–0.030 to 0.015)	.51	–0.046 (–0.075 to –0.018)	.001
Annual renal test	0.843 (0.823 to 0.863)	0.830 (0.810 to 0.850)	–0.013 (–0.030 to 0.004)	.13	0.827 (0.805 to 0.848)	0.845 (0.825 to 0.866)	0.019 (–0.001 to 0.039)	.07	0.032 (0.006 to 0.057)	.02
Hypertension control	0.643 (0.594 to 0.691)	0.628 (0.580 to 0.677)	–0.014 (–0.037 to –0.009)	.22	0.629 (0.581 to 0.677)	0.610 (0.560 to 0.659)	–0.019 (–0.042 to 0.004)	.10	–0.005 (–0.037 to 0.027)	.77
High-risk medication	0.302 (0.276 to 0.328)	0.251 (0.228 to 0.274)	–0.051 (–0.062 to –0.040)	<.001	0.312 (0.286 to 0.339)	0.238 (0.216 to 0.260)	–0.074 (–0.088 to –0.061)	<.001	–0.023 (–0.040 to –0.006)	.01
Timely mental health referral	0.166 (0.142 to 0.190)	0.178 (0.153 to 0.203)	0.012 (–0.006 to 0.018)	<.001	0.182 (0.157 to 0.208)	0.211 (0.182 to 0.239)	0.028 (0.021 to 0.036)	<.001	0.016 (0.006 to 0.026)	.002
Primary care mental health integrated visit	0.025 (0.006 to 0.043)	0.033 (0.009 to 0.057)	0.008 (0.002 to 0.015)	.01	0.012 (0.003 to 0.021)	0.019 (0.005 to 0.034)	0.008 (0.002 to 0.013)	.01	–0.001 (–0.009 to 0.008)	.045
Hospitalization for ACSC	0.035 (0.028 to 0.041)	0.031 (0.026 to 0.037)	–0.003 (–0.006 to –0.001)	.02	0.033 (0.027 to 0.041)	0.025 (0.021 to 0.030)	–0.008 (–0.011 to –0.005)	<.001	–0.004 (–0.009 to <0.001)	.01

Abbreviations: ACSC, ambulatory care–sensitive condition; CoEPCE, Centers of Excellence in Primary Care Education; HbA_1c_, hemoglobin A_1c_.

^a^ Results from logistic mixed effects models, with adjustment for age, gender, race/ethnicity, Elixhauser comorbidity score, and years of VA care, with site as a random effect.

**Figure 2.**
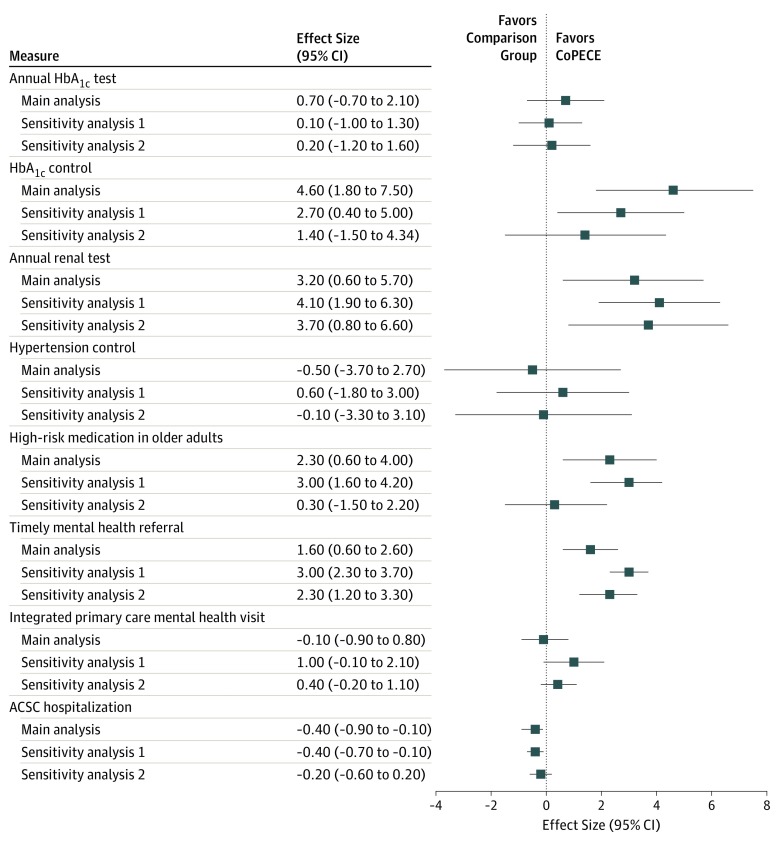
Association of the Centers of Excellence of Primary Care Education (CoEPCE) With Changes in Quality Measures The intervention group consisted of patients cared for by CoEPCE resident clinicians in CoEPCE sites. The 3 comparison groups were as follows: (1) main analysis, patients cared for by non-CoEPCE resident clinicians from non-CoEPCE clinics; (2) sensitivity analysis 1, patients cared for by attending clinicians in the same clinics where CoEPCE trainees practiced; and (3) sensitivity analysis 2, patients cared for by resident clinicians from CoEPCE sites who did not participate in the CoEPCE initiative. Results are presented as effect sizes, which refer to absolute percentage point changes, with 95% CIs. Measures of high-risk medication use and hemoglobin A_1c_ (HbA_1c_) control have been reversed so that the direction that favors intervention vs comparison is consistent across all measures. ACSC indicates ambulatory care–sensitive condition.

A summary of sensitivity analyses is presented in Figure 2. Measures of high-risk medication use and HbA_1c_ control were reversed so that the direction that favors intervention vs comparison is consistent across all measures. For most outcomes, estimates of the association of CoEPCE training with outcomes in the main analysis were the same in direction and similar in magnitude as in sensitivity analyses with alternative comparison groups. For example, in all analyses, we observed improvements in annual renal testing among patients with diabetes (main analysis: effect size, 3.20; 95% CI, 0.60 to 5.70; *P* = .03; sensitivity analysis 1: effect size, 4.10; 95% CI, 1.90 to 6.30; *P* < .001; sensitivity analysis 2: effect size 3.70; 95% CI, 0.80 to 6.60; *P* = .01) and timely mental health referral (main analysis: effect size, 1.60; 95% CI, 0.60 to 2.60; *P* < .001; sensitivity analysis 1: effect size, 3.00; 95% CI, 2.30 to 3.70; *P* < .001; sensitivity analysis 2: effect size 2.30; 95% CI, 1.20 to 3.30; *P* < .001), but we did not observe a difference in annual HbA_1c_ testing among patients with diabetes (main analysis: effect size, 0.70; 95% CI, −0.70 to 2.10; *P* = .03; sensitivity analysis 1: effect size, 0.10; 95% CI, −1.00 to 1.30; *P* = .03; sensitivity analysis 2: effect size, 0.20; 95% CI, 1.20 to 1.60; *P* = .77) or hypertension control (main analysis: effect size, −0.50; 95% CI, −3.70 to 2.70; *P* = .56; sensitivity analysis 1: effect size, 0.60; 95% CI, −1.80 to 3.00; *P* < .001; sensitivity analysis 2: effect size, −0.10; 95% CI, −3.30 to 3.10; *P* = .96). Some outcomes that showed improvement in the main analysis did not reach significance in 1 of 2 sensitivity analyses, such as poor HbA_1c_ control (sensitivity analysis 2: effect size, 1.40; 95% CI, −1.50 to 4.34; *P* = .33) and prescription of a high-risk medication among patients 65 years and older (sensitivity analysis 2: effect size, 0.30; 95% CI, −1.50 to 2.20; *P* = .73). Complete results of sensitivity analyses are included in eTable 3 and eTable 4 in the Supplement.

## Discussion

Prior evaluations of the association of IPE with clinical outcomes were challenged by small sample sizes and limited time frames.^30^ In this evaluation of a large, multisite IPE initiative in VA primary care, we found several notable results. First, we saw an association with modest improvements in quality of care measures, such as annual renal testing and HbA_1c_ control among patients with diabetes and the prescription of high-risk medications among patients 65 years or older. Some measures that improved, such as HbA_1c_ control, depended on patient engagement and participation in care, not simply on a change in clinician behavior, demonstrating change at multiple levels. Additionally, we observed an association with reductions in hospitalizations for ACSCs. Of note, we did not observe any changes that did not favor the CoEPCE initiative, and these findings were robust across sensitivity analyses with alternative comparison groups.

In recent years, numerous large-scale primary care reform efforts, such as patient-centered medical home initiatives, have been designed to improve primary care quality through the implementation of interprofessional teams. While some interventions have changed practice culture, increased practice capacity for change,^31^ and improved patient care,^32^ evaluations of other programs have shown little to no effect on patient-level quality measures.^33,34^ Studies of the implementation of VA PACT demonstrated better performance on clinical quality measures. Nelson et al^35^ found that sites that had more effectively implemented PACT compared with sites that less effectively implemented PACT had better HbA_1c_ control (absolute difference, 2.2%; *P* = .04) and fewer hospitalizations for ACSCs. The CoEPCE initiative was designed to complement the implementation of VA PACT by giving trainees skills to work in interprofessional primary care teams. In our work, we demonstrated changes in clinical quality measures of similar magnitude to effective PACT implementation,^36^ suggesting that a possible mechanism for the association of the CoEPCE initiative with outcomes could be through improved implementation of PACT. This demonstrates that educational initiatives could work synergistically with delivery system reform efforts and that moving reform efforts upstream to train future clinicians in interprofessional practices may have downstream effects on quality of care.

Several recent studies suggest that the quality of care in physician training sites is associated with the future quality of care delivered by physicians who trained there.^37,38^ Our work contributes to this literature by suggesting that teaching environments were associated with patient outcomes. Further work is needed to understand what features of clinical learning environments, outside of interprofessional care, may affect clinical outcomes.^3^

The Kirkpatrick model is a common model used in education to evaluate the effectiveness of learning interventions. It describes effect on the 4 following levels: reaction, learning, behavior, and results.^39^ Evaluations of educational interventions often focus on proximal outcomes, such as learner reaction, engagement, or competency demonstration. Questions regarding how the educational intervention affected care are often left unanswered because obtaining patient-level clinical outcomes for trainees can be a complex, costly process. Our work demonstrated the feasibility of an observational study of an educational intervention’s association with clinical outcomes. Additionally, it highlighted the importance of making explicit associations between education and clinical improvement not just for quality of care purposes but also to assess trainee learning. It is essential to examine clinical outcomes in the design of future educational interventions as trainee portfolios continue to broaden beyond competency achievement and could potentially include assessments of the learning’s effect on clinical outcomes. The improvement of information technology and data availability should allow for such a learning and systems improvement approach with more rapid, agile quantitative analyses providing clinicians, educators, and evaluators with important insights into the consequences of their work as it evolves.

Beyond integrating the clinical education of interdisciplinary trainees, specific clinical and educational innovations performed at CoEPCE sites may have affected clinical outcomes. Some specifically augmented team-based care practices. For example, multiple innovations focused on interprofessional panel management,^40^ interprofessional case conferences,^41^ and the creation of physician and NP dyads that shared care for patient panels.^42^ Several CoEPCE sites developed the PACT Interprofessional Care Update, an interprofessional case conference focused on improving the care of patients at high risk of hospitalization or death. In these conferences, trainees identified patients with high risk and codeveloped proactive care plans with specific action items assigned to trainees of different disciplines.^40,43^ Another site innovation, the initiative to minimize pharmaceutical risk in older veterans, targeted older patients on more than 10 medications with a group visit run by a trainee facilitator, followed by a clinic visit that included comprehensive medication reconciliation.^44^ One site developed a novel NP residency program, consisting of a full-time 12-month clinical training position that bridged NP training to professional practice.^45^ It included education on managing a primary care panel of patients, didactic and workplace learning about topics relevant to primary care practice, and a shared continuity patient panel. This NP residency was later adopted by other sites. Outside of specific interventions, specific teaching on interprofessional care principles and increased interactions among interprofessional trainees may have helped trainees to understand each other’s roles and responsibilities, improve clinical confidence, and develop a group identity based on mutual understanding and trust.^46^ These improved relationships and stronger team identity could facilitate the delivery of high-quality care.

### Limitations

Our work has limitations. First, as an observational study, unobserved characteristics might affect our findings. Specifically, unmeasured differences between sites, such as differences in PACT implementation, could affect our main analysis. However, if our estimates of the association of the CoEPCE initiative with clinical outcomes were associated with site differences over time, we would not expect the use of comparison groups drawn from CoEPCE sites to yield similar results. Second, our effect sizes were modest, and we considered results significant at *P* < .05. Third, while we observed an overall association of the CoEPCE initiative with outcomes, our study did not examine differences in intervention approach, implementation, and context that may have made the CoEPCE initiative successful. Ongoing mixed-methods research from CoEPCE sites, Interprofessional Learning and Practice Partnered Evaluation Center, and OAA may provide further insights on key mechanisms of the CoEPCE initiative.

## Conclusions

In this study, we found that a large, multisite, IPE initiative in VA primary care academic clinics was associated with improved outcomes for patients cared for by interprofessional trainees. This finding suggests that primary care should include a focus on improving clinical learning environments and engaging multiple professions in interdisciplinary education to improve and transform care.

## Notes

Supplement. **eMethods.** Margin Calculations **eTable 1. **Parallel Trends Tests for Main Analysis **eTable 2. **Results from Difference-in-Differences Analysis of Outcomes Without Parallel Trends in the Preintervention Period, External Trainee Comparison Group **eTable 3. **Sensitivity Analysis 1, Changes in Quality of Care Measures Among Patients of CoEPCE Resident Clinicians and Patients of Attending Clinicians at CoEPCE Sites Before and After Initiative Launch **eTable 4. **Sensitivity Analysis 2, Quality of Care Measures Among Patients of CoEPCE Clinician Trainees and Patients of Non-CoEPCE Resident Clinicians at CoEPCE Sites After Initiative Launch (July 2011) at 3 CoEPCE Sites
