# Supplementary material for: Association of a Multisite Interprofessional Education Initiative With Quality of Primary Care
Source: JAMA Netw Open. 2019 Nov 20;2(11):e1915943. doi: 10.1001/jamanetworkopen.2019.15943 (PMC6902823; doi:10.1001/jamanetworkopen.2019.15943)
Supplement: Supplement. — eMethods. Margin Calculations eTable 1. Parallel Trends Tests for Main Analysis eTable 2. Results from Difference-in-Differences Analysis of Outcomes Without Parallel Trends in the Preintervention Period, External Trainee Comparison Group eTable 3. Sensitivity Analysis 1, Changes in Quality of Care Measures Among Patients of CoEPCE Resident Clinicians and Patients of Attending Clinicians at CoEPCE Sites Before and After Initiative Launch eTable 4. Sensitivity Analysis 2, Quality of Care Measures Among Patients of CoEPCE Clinician Trainees and Patients of Non-CoEPCE Resident Clinicians at CoEPCE Sites After Initiative Launch (July 2011) at 3 CoEPCE Sites [file jamanetwopen-2-e1915943-s001.pdf]

Supplementary Online Content

Edwards ST, Hooker ER, Brienza R, et al. Association of a multisite interprofessional education initiative with quality of primary care. *JAMA Netw Open*. 2019;2(11):e1915943. doi:10.1001/jamanetworkopen.2019.15943

eMethods. Margin Calculations

eTable 1. Parallel Trends Tests for Main Analysis

eTable 2. Results from Difference-in-Differences Analysis of Outcomes Without Parallel Trends in the Preintervention Period, External Trainee Comparison Group

eTable 3. Sensitivity Analysis 1, Changes in Quality of Care Measures Among Patients of CoEPCE Resident Clinicians and Patients of Attending Clinicians at CoEPCE Sites Before and After Initiative Launch

eTable 4. Sensitivity Analysis 2, Quality of Care Measures Among Patients of CoEPCE Clinician Trainees and Patients of Non-CoEPCE Resident Clinicians at CoEPCE Sites After Initiative Launch (July 2011) at 3 CoEPCE Sites

This supplementary material has been provided by the authors to give readers additional information about their work.

© 2019 Edwards ST et al. *JAMA Network Open.*

eMethods. Margin Calculations

We used Stata's margins command to obtain predicted probabilities in order to make our results more tangible than model coefficients to readers. Predicted probabilities are also referred to as margins, predictive margins, or adjusted predictions. We can obtain the predicted probability of an outcome in each of four groups (CoEPCE vs. non-CoEPCE; pre- vs. post-periods), given each patient's actual observed values for their covariates. These probabilities are averaged to give us the predicted probabilities for each group (Table 2).

Average marginal effects allow us to show how the adjusted predictions for the pre-period differ from the adjusted predictions at the post-period, by group. Using Stata's margins dydx option, we compared the predicted probability of each person's time period fixed to the pre-period, averaged, vs the predicted probability each person's time period fixed to the post-period, averaged. All other independent variable values were left as is. These predictions were stratified by group, so we could obtain the difference in the probabilities between the time periods for each group. Lastly, we obtained the difference in these two change values, giving us the difference-in-differences value and 95% CIs.

Reference: Williams, R. Using the margins command to estimate and interpret adjusted predictions and marginal effects. The Stata Journal (2012) 12, Number 2, pp. 308–331

eTable 1. Parallel Trends Tests for Main Analysis

	Parallel trend
	p
Annual A1c	0.746
HbA1c poor control	0.853
Annual renal test (microalbumin)	0.943
Hypertension control	0.941
High risk medication in older adults	0.950
Timely mental health referral	0.070
Primary Care Mental Health Integrated Visit	0.969
Hospitalization for ACSC	0.664

© 2019 Edwards ST et al. *JAMA Network Open.*

eTable 2. Results from Difference-in-Differences Analysis of Outcomes Without Parallel Trends in the Preintervention Period, External Trainee Comparison Group

	Patients of non CoEPCE clinician trainees (Comparison)				Patients of CoEPCE Trainees (Intervention)				Difference in Differences		Parallel trends
	2008-2010	2011-2014	Difference	p	2008-2010	2011-2014	Difference	p	Diff	p	p
# Primary Care Visits	3.605 (3.332, 3.877)	3.546 (3.278, 3.813)	-0.059 (-0.101, -0.017)	0.006	3.777 (3.492, 4.062)	3.741 (3.458, 4.023)	-0.036 (-0.084, 0.012)	0.144	0.023 (-0.040, 0.086)	0.424	0.050
ED Visit	0.401 (0.355, 0.447)	0.384 (0.339, 0.430)	-0.017 (-0.025, -0.009)	<0.01	0.405 (0.359, 0.452)	0.351 (0.307, 0.395)	-0.054 (-0.063, -0.045)	<0.01	-0.037 (-0.049, -0.025)	<0.001	0.017
# ED visits	0.928 (0.736, 1.081)	0.850 (0.689, 1.010)	-0.059 (-0.087, -0.031)	<0.01	0.898 (0.728, 1.068)	0.747 (0.605, 0.888)	-0.151 (-0.190, -0.112)	<0.01	-0.092 (-0.139, -0.045)	<0.001	0.022
Hospitalization	0.108 (0.092, 0.124)	0.102 (0.087, 0.117)	-0.006 (-0.011, -0.002)	0.005	0.100 (0.085, 0.115)	0.086 (0.073, 0.099)	-0.014 (-0.019, -0.009)	<0.01	-0.008 (-0.015, -0.001)	0.007	0.047
# Hospitalizations	0.208 (0.170, 0.247)	0.186 (0.152, 0.220)	-0.022 (-0.033, -0.011)	<0.01	0.185 (0.150, 0.219)	0.155 (0.126, 0.184)	-0.030 (-0.040, -0.018)	<0.01	-0.008 (-0.024, 0.008)	0.120	0.013

Results from logistic mixed models or negative binomial mixed models, with adjustment for age, gender, race/ethnicity, Elixhauser comorbidity score, and years of VA care.

© 2019 Edwards ST et al. *JAMA Network Open.*

eTable 3. Sensitivity Analysis 1, Changes in Quality of Care Measures Among Patients of CoEPCE Resident Clinicians and Patients of Attending Clinicians at CoEPCE Sites Before and After Initiative Launch

Predicted probabilities in comparison and COEPCE groups, pre- and post-intervention.

	**Patients of attending physicians at CoEPCE sites (Comparison)**				**Patients of CoEPCE Trainees (Intervention)**				**Diff in diff**	
	2008-2010	2011-2014	Difference	p	2008-2010	2011-2014	Difference	p	Diff	p
Annual A1c	0.955 (0.949, 0.961)	0.960 (0.954, 0.966)	0.005 (<0.001, 0.009)	0.033	0.953 (0.943, 0.962)	0.959 (0.950, 0.968)	0.006 (-0.004, 0.017)	0.033	0.001 (-0.010, 0.013)	0.825
HbA1c poor control	0.208 (0.197, 0.219)	0.231 (0.219, 0.243)	0.023 (0.014, 0.032)	<0.001	0.231 (0.213, 0.249)	0.228 (0.210, 0.246)	-0.003 (-0.025, 0.018)	0.759	-0.027 (-0.050, -0.004)	0.020
Annual renal test (microalbumin)	0.798 (0.778, 0.819)	0.780 (0.758, 0.801)	-0.019 (-0.027, -0.010)	<0.001	0.811 (0.787, 0.834)	0.833 (0.811, 0.855)	0.022 (0.002, 0.043)	0.034	0.041 (0.019, 0.063)	<0.001
Hypertension control	0.663 (0.632, 0.694)	0.639 (0.607, 0.671)	-0.024 (-0.033, -0.014)	<0.001	0.628 (0.593, 0.663)	0.610 (0.574, 0.646)	-0.017 (-0.040, 0.005)	0.123	0.006 (-0.018, 0.030)	0.561
High risk medication in older adults	0.317 (0.294, 0.340)	0.264 (0.243, 0.285)	-0.053 (-0.059, -0.048)	<0.001	0.306 (0.281, 0.330)	0.223 (0.203, 0.243)	-0.083 (-0.096, -0.070)	<0.001	-0.030 (-0.042, -0.016)	<0.001
Timely mental health referral	0.187 (0.161, 0.214)	0.182 (0.156, 0.208)	-0.005 (-0.008, -0.003)	<0.001	0.166 (0.141, 0.191)	0.191 (0.163, 0.218)	0.025 (0.018, 0.031)	<0.001	0.030 (0.023, 0.037)	<0.001
Primary Care Mental Health Integrated Visit	0.020 (-0.004, 0.043)	0.019 (-0.004, 0.041)	-0.001 (-0.003, -0.001)	0.151	0.013 (-0.003, 0.030)	0.022 (-0.004, 0.048)	0.009 (-0.001, 0.019)	0.078	0.010 (-0.001, 0.021)	<0.001
Hospitalization for ACSC	0.023 (0.021, 0.026)	0.020 (0.180, 0.022)	-0.003 (-0.004, -0.002)	<0.001	0.028 (0.025, 0.032)	0.021 (0.019, 0.024)	-0.007 (-0.010, -0.005)	<0.001	-0.004 (-0.007, -0.001)	0.008

© 2019 Edwards ST et al. *JAMA Network Open.*

eTable 4. Sensitivity Analysis 2, Quality of Care Measures Among Patients of CoEPCE Clinician Trainees and Patients of Non-CoEPCE Resident Clinicians at CoEPCE Sites After Initiative Launch (July 2011) at 3 CoEPCE Sites

At CoEPCE sites in Cleveland, West Haven, and Seattle, some resident clinicians participated in the CoEPCE and others did not. Predicted probabilities in comparison and COEPCE groups, post-intervention.

	**Pts of non-CoEPCE trainees** Comparison	**Pts of CoEPCE trainees** Intervention	**Diff**	**p**
Annual A1c	0.960 (0.948, 0.972)	0.962 (0.954, 0.970)	0.002 (-0.012, 0.016)	0.773
HbA1c poor control	0.255 (0.228, 0.282)	0.241 (0.221, 0.261)	-0.014 (-0.434, 0.015)	0.330
Annual renal test (microalbumin)	0.822 (0.783, 0.860)	0.859 (0.830, 0.887)	0.037 (0.008, 0.066)	0.012
Hypertension control	0.604 (0.569, 0.638)	0.603 (0.574, 0.631)	-0.001 (-0.033, 0.031)	0.959
High risk medication in older adults	0.254 (0.229, 0.280)	0.251 (0.229, 0.274)	-0.003 (-0.022, 0.015)	0.730
Timely mental health referral	0.205 (0.179, 0.231)	0.228 (0.201, 0.254)	0.023 (0.012, 0.033)	<0.001
Primary Care Mental Health Integrated Visit	0.022 (0.003, 0.042)	0.026 (0.004, 0.048)	0.004 (-0.002, 0.011)	0.223
Hospitalization for ACSC	0.027 (0.022, 0.031)	0.025 (0.021, 0.029)	-0.002 (-0.006, 0.002)	0.348

CoEPCE – Centers of Excellence in Primary Care Education, ED -emergency department, ACSC ambulatory care sensitive condition.
Results from logistic mixed models, with adjustment for age, gender, race/ethnicity, Elixhauser comorbidity score, and years of VA care.

© 2019 Edwards ST et al. *JAMA Network Open.*
